# Activation of NF-κB/MAPK signaling and induction of apoptosis by salicylate synthase NbtS in *Nocardia farcinica* promotes neuroinflammation development

**DOI:** 10.1128/msystems.00893-24

**Published:** 2024-09-06

**Authors:** Jirao Shen, Xing zhao Ji, Lichao Han, Jiang Yao, Yihe Liang, Min Yuan, Shuai Xu, Zhenjun Li

**Affiliations:** 1 National Key Laboratory of Intelligent Tracking and Forecasting for Infectious Diseases, National Institute for Communicable Disease Control and Prevention, Chinese Center for Disease Control and Prevention, Beijing, China; 2 Department of Pulmonary and Critical Care Medicine, Shandong Provincial Hospital Affiliated to Shandong First Medical University, Jinan, China; 3 School of Laboratory Medicine and Life Sciences, Wenzhou Medical University, Wenzhou, China; Zhejiang University School of Medicine, Hangzhou, Zhejiang, China

**Keywords:** *Nocardia farcinica*, NbtS protein, microglial cells, neuroinflammation

## Abstract

*Nocardia farcinica* can cause a rare, yet potentially fatal, central nervous system infection. NbtS protein may be a key virulence factor in *N. farcinica* infection of the brain. In this study, we investigated the function of the virulence-associated factor NbtS in microglial cells *in vitro* and in infected mice *in vivo*. We explored the interactions between NbtS and microglial cells (BV2 and human microglial clone 3), revealing that NbtS activates the toll-like receptor 4-dependent MyD88-IRAK4-IRAK1 and MAPK/nuclear factor kappa B (NF-κB) pathways, significantly enhancing pro-inflammatory responses as indicated by increased levels of tumor necrosis factor alpha (TNF-α) and interleukin-1β (IL-1β), as measured by ELISA and quantitative PCR. Apoptosis was elevated in these cells, as shown by increased expression of Bax and caspase-3 and decreased Bcl-2 levels. The terminal deoxynucleotidyl transferase dUTP nick end labeling assay also confirmed the occurrence of apoptosis. In vivo, mice infected with an *RS03155*-deficient strain of *N. farcinica* exhibited higher survival rates and reduced brain inflammation, suggesting a pivotal role for the NbtS protein in the pathogenesis of *Nocardia*. Conservation of the *RS03155* gene across *Nocardia* spp. was verified by PCR, and the immunogenic potential of NbtS was confirmed by Western blot analysis using sera from infected mice. These findings suggest that targeting NbtS may offer a novel therapeutic strategy against *Nocardia* infection.

**IMPORTANCE:**

The study presented in this article delves into the molecular underpinnings of *Nocardia farcinica*-induced neuroinflammation. By focusing on the salicylate synthase gene, *RS03155*, and its encoded protein, NbtS, we uncover a pivotal virulence factor that triggers a cascade of immunological responses leading to apoptosis in microglial cells. This research not only enhances our comprehension of the pathogenesis of *Nocardia* infections but also provides a potential therapeutic target. Given the rising importance of understanding host-microbe interactions within the context of the central nervous system, especially in immunocompromised individuals, the findings are of significant relevance to the field of microbiology and could inform future diagnostic and treatment modalities for *Nocardia*-associated neurological disorders. Our work emphasizes the need for continued research into the intricate mechanisms of microbial pathogenesis and the development of novel strategies to combat life-threatening infections.

## INTRODUCTION

*Nocardia*, an aerobic actinomycete and Gram-positive branched bacillus, exhibits partial acid resistance and facultative intracellular growth. It is globally distributed in soil, plants, and water (1). *Nocardia* invades various host tissues and organs (2), showing a particular affinity for the central nervous system (CNS) and causing cerebral nocardiosis. Cerebral nocardiosis, a rare yet potentially fatal central nervous system infection, must be considered when diagnosing brain abscesses in both immunocompromised and immunocompetent individuals (3). The atypical clinical manifestations of cerebral nocardiosis make early diagnosis challenging, potentially delaying treatment.

While some research has documented how *Nocardia* induces disease, little focuses on cerebral nocardiosis. For example, the cell wall proteins Mce1C and Mce1D of *Nocardia* stimulate mouse mononuclear macrophages to secrete inflammatory cytokines, aiding the strain’s invasion of HeLa cells (4). *Nocardia cyriacigeorgica*’s HBHA may facilitate colonization of various organs during infection, stimulating inflammatory cytokines and modulating the innate immune response (5). Additionally, *Nocardia* produces various siderophores; some may damage host tissues. *Nocardia farcinica* was used to infect A549 cells, with bacterial RNA collected at 1, 3, and 6 h post-infection for transcriptomic analysis. Results showed significant upregulation of genes involved in non-ribosomal peptide biosynthesis of siderophores, including *RS03155*. Knocking out this gene significantly increased survival rates in infected mice and alleviated the neurological symptoms of *N. farcinica* infection (6). However, their studies were largely phenotypic and did not determine whether the neurological symptoms caused by *Nocardia* infection are due to direct neuronal effects or primarily mediated by microglial-induced neuroinflammation. The specific cellular and molecular mechanisms by which NbtS damages host cells and potentially modulates the host’s immune response remain unclarified, and the expression of NbtS protein across different *Nocardia* spp. has not been explored.

Innate immunity, the body’s first defense against microbial pathogens, activates through specific recognition systems. The brain’s innate immune system is regulated by various factors. Microglia, the central nervous system’s macrophages, form a crucial defense against brain infections (7). When microbial pathogens penetrate the blood-brain barrier and invade the brain, microglia activate in response to various stimuli such as pathogen-associated molecular patterns (PAMPs), damage-associated molecular patterns (DAMPs), and pro-inflammatory mediators, producing cytokines, chemokines, and reactive species (8). The expression and function of key molecules in the toll-like receptor 4 (TLR4)-dependent MyD88-IRAK4-IRAK1 signaling axis influence these activities. Collectively, these substances help eliminate invading microbes. Special microglial receptors bind to pathogenic DAMPs, triggering increased oxidative stress and releasing transcription factors nuclear factor kappa-light-chain enhancer of activated B-cell (NF-κB) and MAPK, which result in neuroinflammation (9, 10). Moreover, this pro-inflammatory activation can promote apoptosis in microglia, mediated by both intrinsic and extrinsic death receptors (11). In *Mycobacterium tuberculosis*, some enzymes are capable of activating microglial cells to elicit inflammatory responses (12). Similarly, as a member of the actinobacteria, we aim to investigate whether the NbtS protein in *N. farcinica* possesses this function.

Research on *Nocardia*’s virulence factors in brain infections is limited, underscoring the need for detailed knowledge of its molecular interactions with host cells. In this study, we cloned and expressed recombinant NbtS protein and constructed an *RS03155* deletion mutant (Δ*RS03155*) in *N. farcinica* to evaluate the effects of the *RS03155* gene on brain infections. We then analyzed the interactions between NbtS and microglia cells and explored how nbtS regulates the innate immune system. Our findings suggest that *RS03155* is a key virulence gene in *N. farcinica* and that NbtS, which is prevalent in *Nocardia*, may play a critical role in its infections, offering potential prevention and control strategies. Additionally, NbtS was shown to enhance tumor necrosis factor alpha (TNF-α) and interleukin-1β (IL-1β) production in microglia, activate the MAPK/NF-κB signaling pathway, and induce intrinsic apoptosis. Our findings offer insights into the mechanisms of *Nocardia* infection and contribute to developing prevention and control strategies.

## RESULT

### The critical role of *RS03155* gene in *N. farcinica* brain infection severity, lethal outcomes, and neuroinflammation in mice

**Fig 1:**
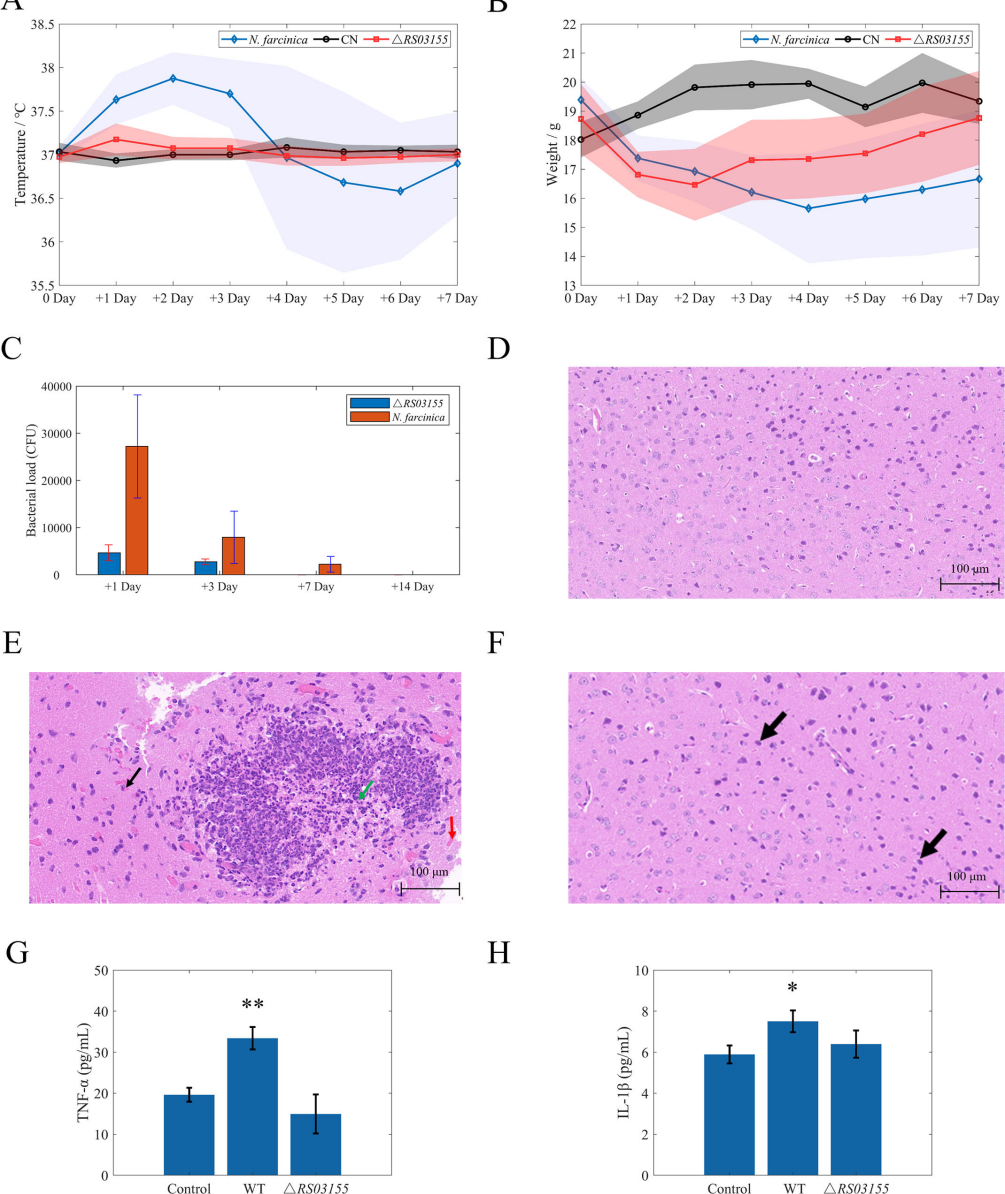
Effects of *RS03155* gene on the pathogenicity of *N. farcinica*. Changes in body temperature (**A**), body weight (**B**), and brain tissue bacterial load (**C**) following infection of mice with the *N. farcinica*, *N. farcinica* Δ*RS03155* mutants (Δ*RS03155*), and phosphate-buffered saline (the control group [CN]) infections. (D–F) Histopathological staining results of brain tissue 14 days post-infection. (G and H) Inflammatory cytokine profile of brain tissue 14 days post-infection. Significance is denoted by * for *P* < 0.05 and ** for *P* < 0.01, compared to the control group.

Following infection with both the wild-type and *RS03155* deletion strains, mice infected with the wild-type strain showed a rapid decline in body weight and significant increase in body temperature within 1–4 days post-infection (dpi) (Fig. 1A). Conversely, mice infected with the deletion strain exhibited only slight decreases in body weight and minimal temperature fluctuations during the first 2 dpi. (Fig. 1B). Assessment of bacterial load in brain tissues from 1 to 14 dpi revealed significantly higher bacterial burden in mice infected with the wild-type strain compared to those infected with the *RS03155* knockout strain (Fig. 1C). Histopathological analysis showed focal necrosis of cortical neurons in mice infected with the wild-type strain, characterized by nuclear fragmentation, significant granulocyte infiltration, and vascular congestion. The knockout group displayed indistinct nuclear and cytoplasmic details (Fig. 1D through F). In addition, after 7 days of infection with the wild-type strain, the concentrations of the inflammation cytokines TNF-α and IL-1β in the brain tissue of mice showed a significant increase (Fig. 1H). It is evident that the *RS03155* gene plays a critical role in enhancing the virulence, mortality, and inflammatory response in the brains of mice infected with the wild-type *N. farcinica*.

### Analyzing the *RS03155* gene for conservation and its NbtS protein for antigenic properties

**Fig 2:**
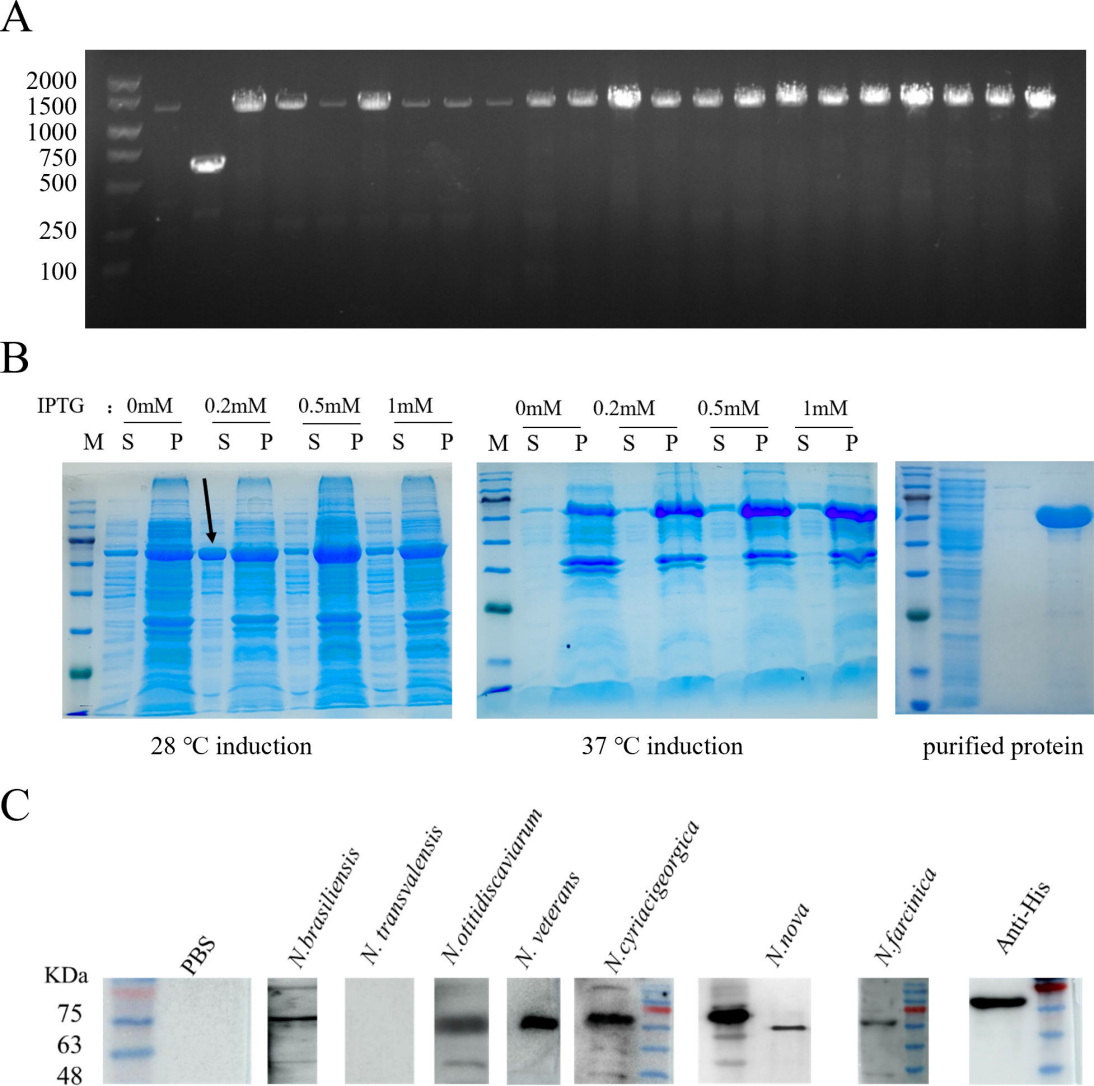
(**A**) *RS03155* gene carrying status of clinical strains of *N. farcinica*. (**B**) SDS-PAGE analysis of NbtS protein expression. Arrows indicate optimal induction results. (**C**) Reactivity of NbtS protein with anti-NbtS sera was determined by Western blot. P, precipitate; S, supernatant.

PCR screening of 20 clinical *N. farcinica* strains showed that all contained gene fragments of the same size as those in the IFM10152 strain (Fig. 2A), indicating high conservation of the *RS03155* gene. Antigenic epitope prediction using Ellipro (http://tools.iedb.org/ellipro/) identified 14 potential antigenic determinants, suggesting its potential for strong antigenicity.

DNA sequencing confirmed the successful construction of the pET32a-NbtS recombinant vector. Protein expression was analyzed by SDS-PAGE following isopropyl β-D-1-thiogalactopyranoside (IPTG) induction. Optimal induction of NbtS protein was achieved with 0.2 mM IPTG at 28°C, primarily in the supernatant, showing a single band upon purification (Fig. 2B). The recombinant His-NbtS protein was identified using an anti-His antibody. Western blot analysis showed that the NbtS protein was able to react with serum antibodies from mice infected with *N. farcinica*, *Nocardia brasiliensis*, *Nocardia otitidiscaviarum*, *Nocardia veterans*, *N. cyriacigeorgica*, and *Nocardia nova* but did not react with control serum and *Nocardia transvalensis* (Fig. 2C). This indicates that the NbtS protein is ubiquitously present in *Nocardia* spp.

### NbtS protein reduces cell viability and enhances lactate dehydrogenase release in BV2 and human microglial clone 3 cells

**Fig 3:**
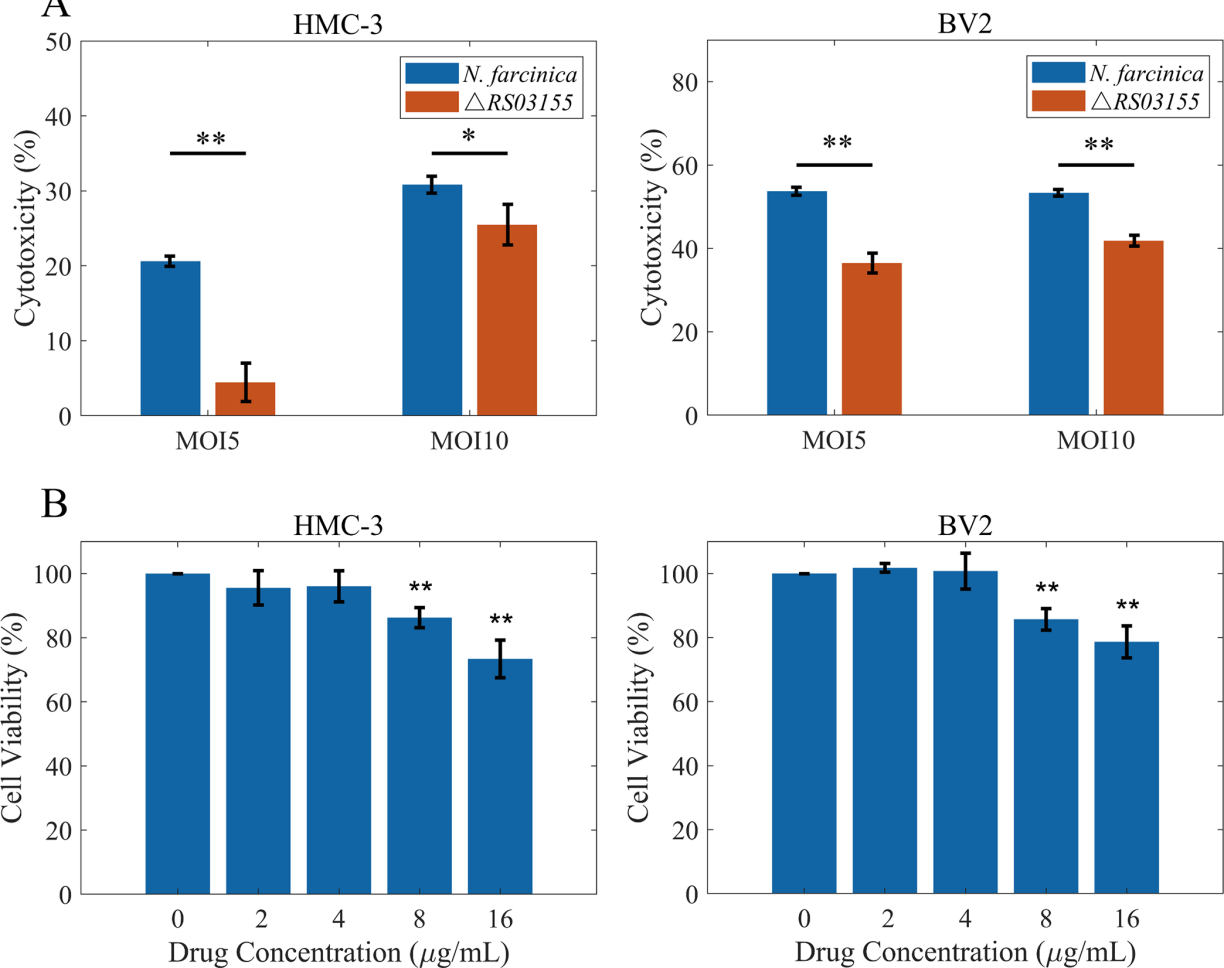
(**A**) The effect of the *RS03155* gene in *N. farcinica* on cytotoxicity in BV2 and HMC-3 cells. Significance is denoted by * for *P* < 0.05 and by ** for *P* < 0.01. (**B**) The impact of the NbtS protein on the viability of BV2 and HMC-3 cells. Significance is denoted by * for *P* < 0.05 and by ** for *P* < 0.01, compared to the control group.

Lactate dehydrogenase (LDH) release assay revealed that the wild-type *N. farcinica* strain significantly increased LDH release in human microglial clone 3 (HMC-3) and BV2 cells, while the *RS03155* knockout strain induced markedly less LDH release (Fig. 3A). The results suggest that the *RS03155* gene may significantly contribute to the virulence of *N. farcinica*. The *RS03155* gene is likely to play a role in the neuropathological changes induced by *N. farcinica*, affecting NbtS protein treatment significantly reduced viability in HMC-3 and BV2 cells (Fig. 3B). This implies that NbtS may contribute to *Nocardia*’s capacity to cause cellular damage and potentially modulate host immune responses during infection.

### NbtS stimulated the production of TNF-α and IL-1β in BV2 and HMC-3 cells

**Fig 4:**
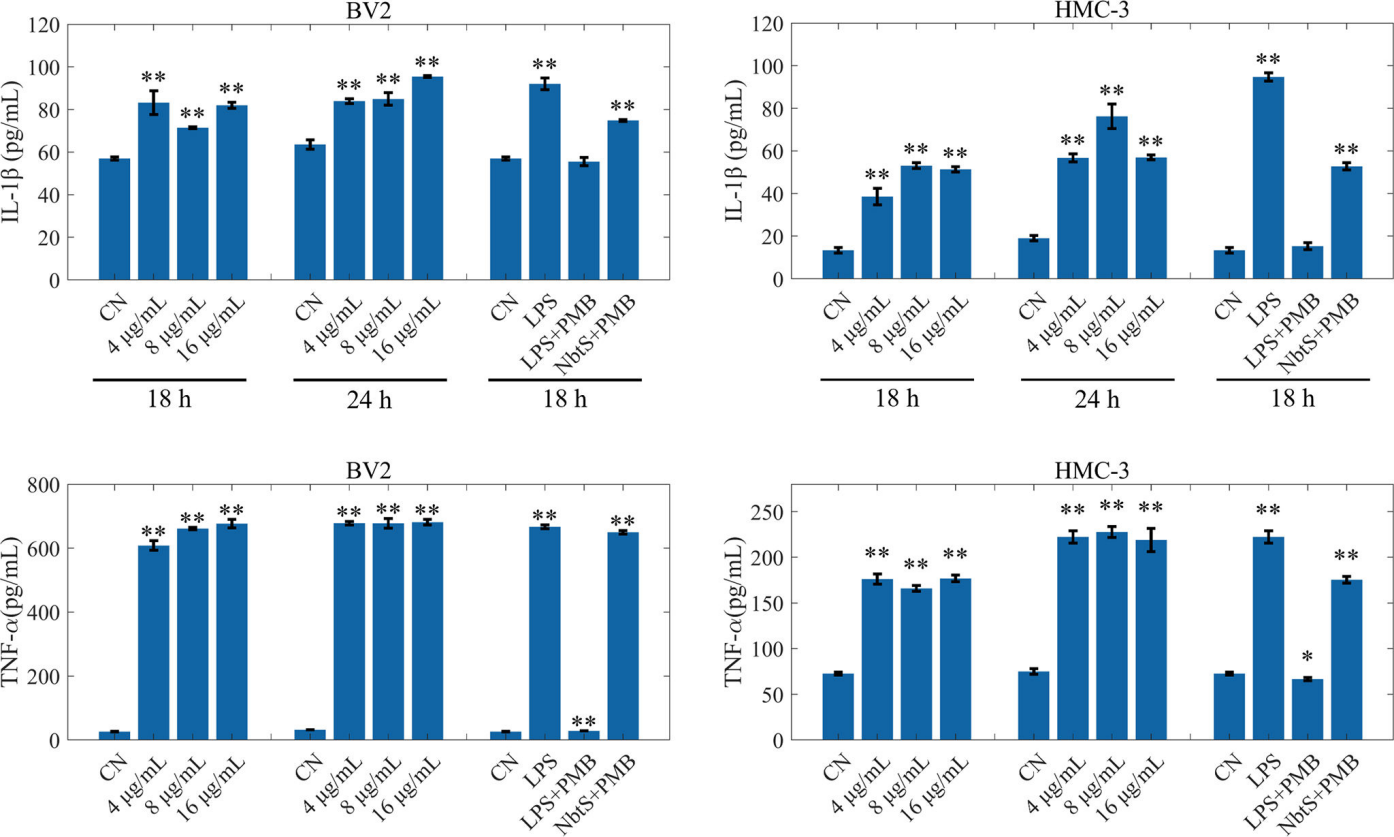
Changes in IL-1β and TNF-α levels in BV2 and HMC-3 cells stimulated with different concentrations of NbtS protein at 18 and 24 h. Significance is denoted by * or **, indicating a *P* value of <0.05 or <0.01 when compared to the control group (CN).

To assess if NbtS stimulates inflammatory responses in the brain, HMC-3 and BV2 cells were treated with NbtS at 4, 8, and 16 µg/mL for 18 and 24 h. Culture supernatants were collected, and TNF-α and IL-1β levels were measured via ELISA (Fig. 4). To rule out lipopolysaccharide (LPS) contamination effects, NbtS was pre-treated with polymyxin B using LPS as a positive control. The induction of TNF-α and IL-1β expression by the NbtS protein, independent of LPS contamination, suggests that NbtS enhances the inflammatory and damaging effects of *N. farcinica* infections in the brain. This may be associated with neuroinflammation, cellular damage, and immune modulation.

### NbtS activates inflammatory signaling pathways in BV2 and HMC-3 cells

**Fig 5:**
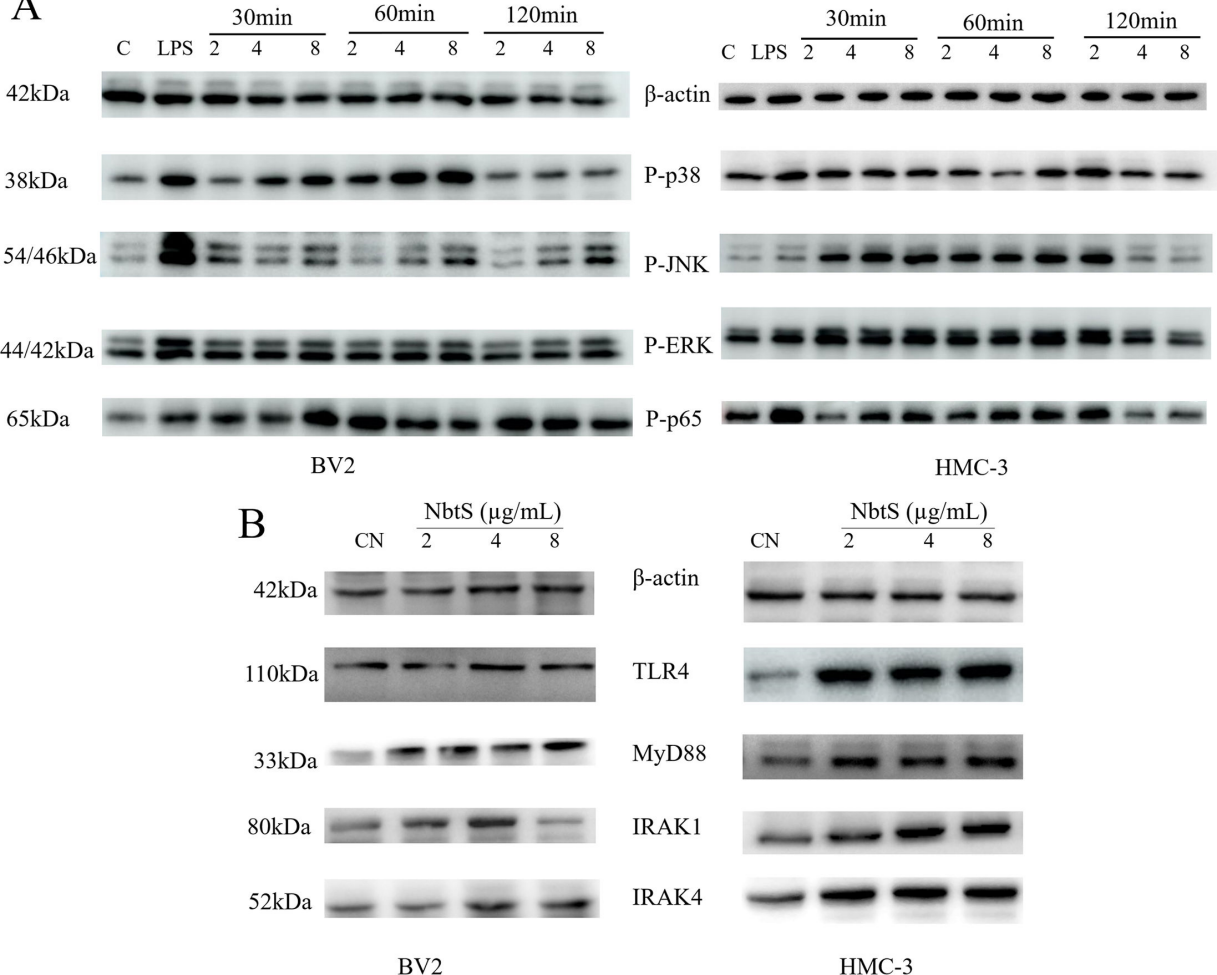
(**A**) Western blot analysis of the expression levels of phosphorylation of ERK, p38, JNK and p65 in BV2 and HMC-3 cells stimulated with different concentrations of NbtS protein at specified time points. (**B**) Western blot analysis of the expression levels of IRAK4, IRAK1, MyD88, and TLR4 in BV2 and HMC-3 cells stimulated with different concentrations of NbtS protein.

In this study, the impact of NbtS on the immune responses of BV2 and HMC-3 cells was investigated. We assessed the phosphorylation levels of key signaling molecules such as p-p38, p-JNK, p-ERK, and p-p65 using Western blot analysis due to their central roles in cellular stress and inflammatory responses. BV2 and HMC-3 cells were stimulated with NbtS at concentrations of 2, 4, and 8 µg/mL. Stimulation with NbtS resulted in the phosphorylation of p38, JNK, ERK1/2, and p65 at 30 and 60 min post-treatment. Two hours after NbtS stimulation, the phosphorylation levels of p38, JNK, ERK1/2, and p65 started to return to baseline (Fig. 5A). Phosphorylation was more pronounced at different time intervals with an induction of 8 µg/mL. This indicates that the immune response in microglial cells to NbtS stimulation is both time dependent and dose dependent. Our results suggest the potential activation of inflammation-related signaling pathways by NbtS. After assessing phosphorylation levels, we evaluated the expression levels of IRAK4, IRAK1, MyD88, and TLR4. These molecules are key components in recognizing PAMPs and regulating inflammatory responses. Upon stimulating BV2 and HMC-3 cells with various concentrations of NbtS for 1 h, we noted changes in the expressions of IRAK4, IRAK1, MyD88, and TLR4 (Fig. 5B), coinciding with the upregulation of p-p38, p-JNK, p-ERK, and p-p65. In summary, this study demonstrates that the NbtS protein significantly modulates immune responses in BV2 and HMC-3 cells by inducing the phosphorylation of key inflammatory signaling molecules and altering the expression of critical immune response components, suggesting its role in the regulation of inflammatory pathways.

### NbtS stimulates cytokine production via activation of the MAPK and NF-κB signaling pathways

**Fig 6:**
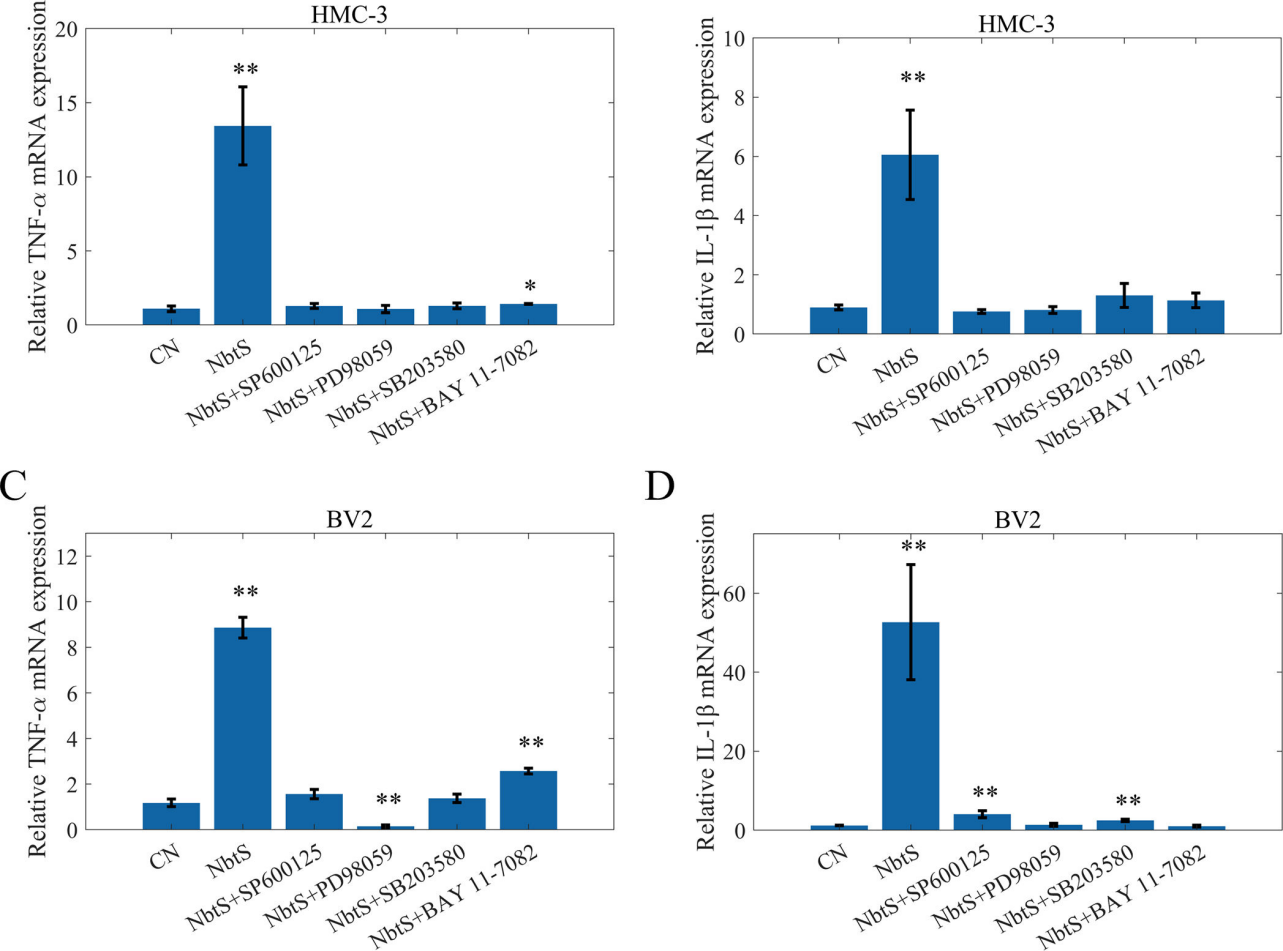
NbtS-mediated cytokine expression was dependent on ERK1/2, JNK, and NF-κB. BV2 and HMC-3 cells were pre-incubated with 20 µM PD98059, 20 µM SP60012, 10 µM SB203580, or 10 µM BAY 11–7082 for 1 h and then stimulated with Nfa34810 (8 µg/mL). After incubation, total RNA was extracted, and (**A**) HMC-3 TNF-α, (**B**) HMC-3 IL-1β, (**C**) BV2 TNF-α, (**D**) BV2 IL-1β expression level was measured using quantitative PCR method. Significance is denoted by **, indicating a *P* value less than 0.01 when compared to the control group.

To ascertain the roles of JNK, p38, ERK1/2, and NF-κB in the TNF-α and IL-1β production induced by NbtS, HMC-3 and BV2 cells were pre-treated with 20 µM PD98059 (a MEK inhibitor), 20 µM SP600125 (a JNK inhibitor), 10-µM SB203580 (a p38 MAPK inhibitor), or 10 µM BAY 11–7082 (an NF-κB inhibitor) for 1 h, followed by the addition of NbtS (8 µg/mL). Expression levels of TNF-α and IL-1β were quantified via quantitative PCR after 18 h. As illustrated in the figures, inhibitors of ERK1/2, JNK, and NF-κB significantly suppressed the expression of TNF-α and IL-1β (Fig. 6A through D). These results indicate that the ERK1/2, JNK, p38, and NF-κB signaling pathways play crucial roles in the neuroinflammatory response induced by NbtS.

### The role of the *RS03155* gene in *N. farcinica*-induced apoptosis in microglial cells

**Fig 7:**
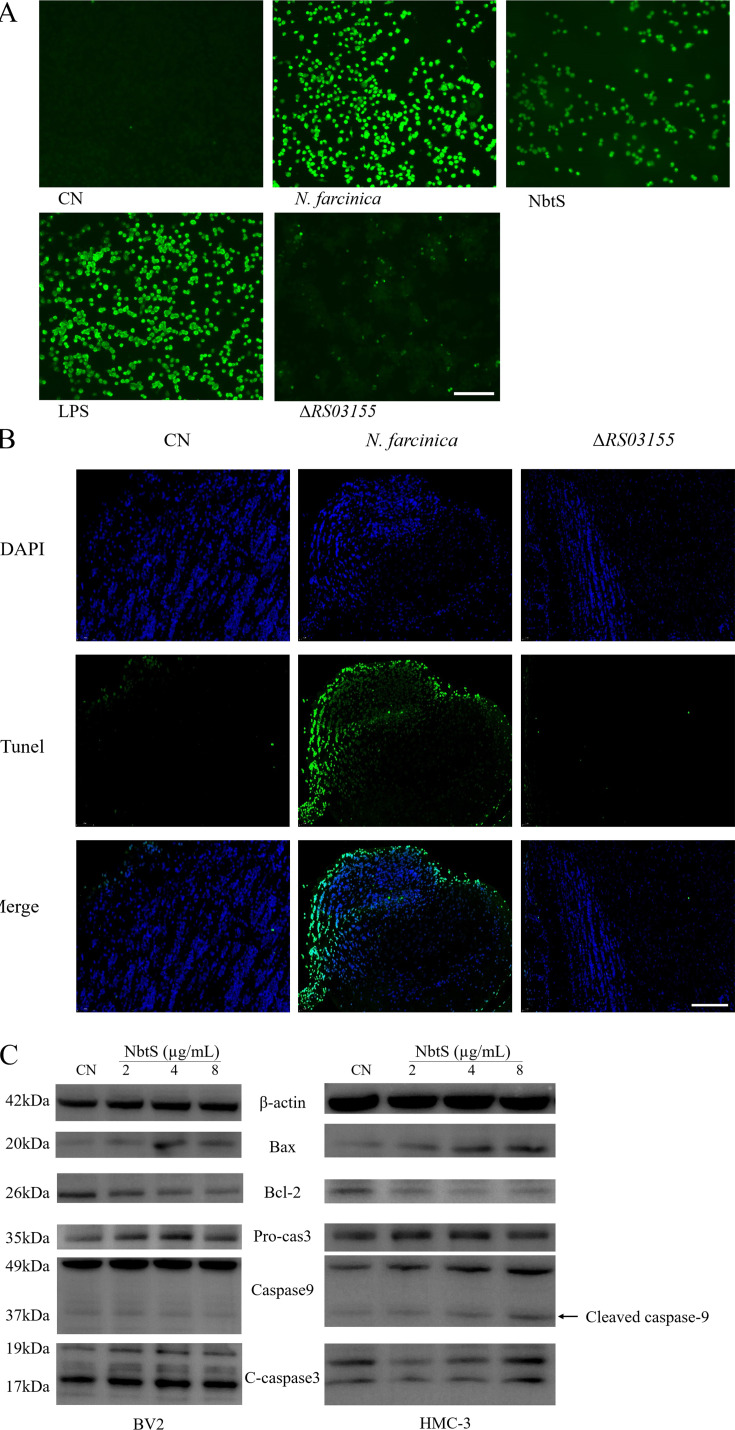
The role of the *RS03155* gene in *N. farcinica*-induced apoptosis in microglial cells. (**A**)The TUNEL-positive cells at 12 h after BV2 cells were treated for with wild-type *N. farcinica*, the *RS03155* mutant strain, NbtS protein, and LPS were measured by TUNEL kit. Scale bar 25 µm. (**B**) TUNEL fluorescent staining representative images of each group. The green fluorescence shows TUNEL-positive cells (apoptotic cells). Scale bar 100 µm. (**C**) NbtS protein induced increased the expression of the Bax, pro-caspase-3, and caspase-9; cleaved caspase-3c; and downregulated the expression of Bcl-2 in microglial cells.

The role of the *RS03155* gene in *N. farcinica*-induced microglial apoptosis was assessed using terminal deoxynucleotidyl transferase dUTP nick end labeling (TUNEL) assays and Western blot analysis of apoptosis-related proteins. TUNEL assay revealed that both the wild-type strain and the NbtS protein induced apoptosis in BV2 cells, whereas apoptosis was not significantly observed in the knockout strain group (Fig. 7A). In the mouse infection model, a large number of TUNEL-positive cells were observed in the brains of mice infected with the wild-type strain of *N. farcinica*. In contrast, the number of positive cells was reduced in the *RS03155* knockout strain (Fig. 7B). This finding supports the significant role of the *RS03155* gene in mediating cellular damage and death caused by *N. farcincia*. Western blot analysis demonstrated that, compared to the control group, cells treated with NbtS significantly decreased the Bcl-2:Bax ratio and increased the expression of pro-caspase-3 and caspase-9, ultimately leading to the upregulation of cleaved caspase-3 expression (Fig. 7C). This indicates that NbtS may promote apoptosis by activating endogenous apoptotic pathways, particularly by influencing the mitochondrial-mediated caspase cascade.

## DISCUSSION

Central nervous system nocardiosis presents a challenging infection for clinicians, not only due to its non-specific symptoms and complex imaging findings, primarily affecting individuals with compromised immune systems, but also due to a high mortality rate of up to 35% within a year (13). This mortality rate significantly exceeds the 10% observed in patients with other bacterial brain abscesses (14). The pathogenic mechanisms and virulence factors of brain diseases caused by *Nocardia* remain poorly understood. In our preliminary research, it was discovered that following infection of A549 cells by *N. farcinica*, there was a significant upregulation of genes associated with non-ribosomal peptide synthesis linked to siderophore groups. By constructing a series of *N. farcinica* knockout strains, it was found that mice infected with the Δ*RS03155* strain exhibited a notably higher survival rate. The pathogenic role of NbtS in *N. farcinica* has been confined to phenotypic experiments and has not been explored in depth. NbtS shares functional similarities with MtbI in *M. tuberculosis*, both acting as salicylate synthases. MtbI synthesizes siderophores in *M. tuberculosis*, essential for the bacterium’s survival and pathogenicity (15). Mori and colleagues discovered a novel MbtI inhibitor that demonstrated significant anti-tubercular activity in *M. tuberculosis* cultures (16). The irp1 gene, another salicylate synthase, creates a virulence island in *Escherichia coli*, resulting in severe tissue damage in mice. Knocking out irp1 significantly reduces *E. coli*’s virulence (17).

In this study, *N. farcinica* wild-type strain and *RS03155* knockout strain, respectively, were employed to infect mice. Hematoxylin and eosin staining results indicated that the wild-type strain exhibited greater neurotoxicity and a higher capacity for inflammatory induction. Differences in brain tissue bacterial loads between wild-type and knockout strains suggest the *RS03155* gene crucially impacts the integrity of *N. farcinica*’s disruption of the blood-brain barrier. Consequently, mice infected with the wild-type strain of *N. farcinica* experienced more severe clinical symptoms and brain infections. Although many microbes can invade the bloodstream, few are able to spread to the meninges because the central nervous system compartments are protected by barriers. These barriers maintain neuronal environment homeostasis and protect neuronal tissues from infection. Bloodborne bacteria capable of invading the central nervous system have evolved mechanisms to bypass these barriers (18). The *RS03155* gene likely represents a specific mechanism for *N. farcinica*’s brain invasion.

Conservation analysis of the *RS03155* gene reveals high conservation across various clinical strains of *N. farcinica*, indicating a potentially crucial role in its survival and pathogenicity. This finding aligns with growth curve results, showing slower growth in the *RS03155* gene knockout strain compared to the wild type (see Fig. S1 and S2 in the supplemental material). Generally, in other bacteria, gene conservation is crucial for adaptation to host environments and maintaining pathogenicity. For example, the protease IV gene can be detected in all strains of *Pseudomonas aeruginosa* but absent in non-*P*. *aeruginosa* strains. Mutants deficient in protease IV exhibit reduced corneal toxicity relative to their parent strains (19). All group A *Streptococcus* strains possess one of two variants of the Mga gene. Proteins homologous to the Mga protein occur in other pathogenic streptococcal species. This suggests conservation of the Mga protein and its regulatory functions across streptococcal species, potentially playing similar roles in their pathogenic mechanisms (20). The NbtS protein reacts with antibodies from sera of mice infected with various *Nocardia* spp., indicating cross-species immunogenicity. This has important implications for developing broad-spectrum diagnostic tools and vaccines against *Nocardia* infections. Additionally, all these *Nocardia* spp. can cause brain infections (6), suggesting an association between the NbtS protein and *Nocardia* virulence. These results suggest the potential to develop a diagnostic method for highly virulent *Nocardia* strains based on the NbtS protein. This offers a direction for the rapid diagnosis of cerebral nocardiosis.

Earlier studies have demonstrated that *N. brasiliensis* and *N. farcinica* induce macrophages to produce pro-inflammatory cytokines, including TNF-α, IL-6, and IL-1β (21, 22). The HBHA protein and Nfa34810, linked to *Nocardia* pathogenicity, also trigger inflammatory factor production in macrophage-like cells (5, 22). Therefore, to fully understand their functions, we further analyzed the role of NbtS in the interaction with microglial cells, aiming for deeper insights into the pathogenic mechanisms of *Nocardia*’s brain invasion.

CCK8 results indicated that the NbtS protein from *N. farcinica* has a cytotoxic effect on microglial cells, decreasing cell viability with higher protein concentrations, possibly interfering with cellular functions or triggering apoptotic or necrotic pathways. Additionally, the wild-type strain of *N. farcinica* induces a more significant release of LDH compared to the NbtS knockout strain, indicating that the NbtS protein contributes to the cytotoxicity and virulence of the bacterium. This protein may contribute to the bacterium’s ability to damage host cells and evade immune responses. Previous studies on the virulence factors of *Nocardia* have primarily focused on models outside the central nervous system, resulting in a lack of understanding of the pathogenic mechanisms of cerebral nocardiosis. Therefore, this study further investigates the role of the NbtS protein in brain infection by *N. farcinica*.

Our findings demonstrate that recombinant NbtS protein from *N. farcinica* significantly upregulates the expression of TNF-α and IL-1β in HMC-3 and BV2 cells, with a more pronounced effect at 24 h. The NF-κB and MAPK signaling pathways are critical for neuroinflammation induction (23). The NF-κB pathway is responsible for the transcription of various pro-inflammatory cytokines, chemokines, and adhesion molecules, crucial in diseases with dysregulated inflammatory responses (24). ERK, JNK, and p38 can activate individually or together to stimulate pro-inflammatory cytokine expression (25). Our results suggest rapid phosphorylation of p38, JNK, ERK1/2, and p65 by the NbtS protein. To analyze the role of phosphorylated MAPK and NF-κB in cytokine production, we employed specific inhibitors of p38 (SB203580), ERK1/2 (PD98059), JNK (SP600125), and NF-κB (BAY-117082) to block these kinase cascades. Our findings reveal that these inhibitors suppress TNF-α and IL-1β production, underscoring the NbtS protein’s impact on cytokine production via the MAPK and NF-κB pathways. Phosphorylation of p-p38, p-JNK, p-ERK, and p-p65 represents an immediate cellular response to stimulation by NbtS protein, indicating that NbtS protein initiates intracellular signaling cascades. TLR4, a key receptor in the immune system for recognizing foreign pathogens, upon activation, triggers a series of signaling events, ultimately leading to the activation of the NF-κB and MAPK pathways (26). IRAK4 and IRAK1 are critical kinases within the TLR signaling pathway, while MyD88 serves as an adaptor molecule in the TLR pathway (27). As the primary receptor for detecting bacterial LPS, variations in TLR4 expression may suggest a potential interaction between NbtS protein and TLR4. Based on these findings, it is plausible to hypothesize that NbtS protein may activate the TLR4, which then recruits IRAKs through MyD88, activating the MAPK and NF-κB signaling pathways and thereby promoting inflammatory responses.

Neuroinflammation can lead to apoptosis. TUNEL assay results following stimulation of BV2 cells by *N. farcinica* and NbtS protein suggest that the protein encoded by the *RS03155* gene or its expression products may directly participate in the induction of apoptosis. TUNEL experiments conducted on brain tissues in the mouse infection model further confirm the role of the *RS03155* gene in inducing apoptosis *in vivo*. This may provide a direction for developing new therapeutic strategies, such as targeting this gene through gene editing technologies to mitigate the pathological effects of *N. farcinica* infection. After stimulating microglial cells with NbtS, we observed significant upregulation of the pro-apoptotic proteins bax, caspase-3, caspase-9, and cleaved caspase-3 and a significant downregulation of the protective protein Bcl2. One of the core changes induced by infection is an increase in neurotoxicity caused by inflammatory products (including IL-1β and TNF-α), oxidative stress (including lipid peroxidation), and apoptosis. After stimulating microglial cells with NbtS, we observed significant upregulation of the pro-apoptotic proteins bax, caspase-3, caspase-9, and cleaved caspase-3 and a significant downregulation of the protective protein Bcl2 (28). Increased expression of bax, caspase-3, caspase-9, and cleaved caspase-3 suggests activation of both intrinsic (mitochondrial) and extrinsic (death receptor) apoptotic pathways. Bax is a pro-apoptotic bcl-2 family member that promotes mitochondrial outer membrane permeabilization, leading to cytochrome c release and subsequent activation of caspase-9 and caspase-3, culminating in cell death. The elevated levels of cleaved caspase-3, an active form of caspase-3, further confirm that the execution phase of apoptosis is under way (29). The findings have significant implications for understanding how *N. farcinica* infections contribute to neuroinflammatory and neurodegenerative processes. As the primary immune cells in the CNS, microglial cells play a pivotal role in maintaining neuronal health. Apoptosis of these cells, driven by bacterial proteins such as NbtS, could lead to dysregulated immune responses and exacerbate neuronal damage (30).The activation of NF-κB/MAPK signaling and the subsequent apoptosis of microglial cells in the brain can have significant implications for neurodegenerative diseases. Dysregulated microglial activation and apoptosis can contribute to neuronal damage and the progression of diseases like Alzheimer’s disease, Parkinson’s disease, and multiple sclerosis (31).

Currently, through both *in vivo* and *in vitro* experimental models, we have confirmed the role of the *RS03155* gene in inducing brain apoptosis following *N. farcinica* infection, as evidenced by TUNEL assays. Additionally, in the *in vitro* model, we have elucidated the molecular mechanisms by which NbtS induces apoptosis. However, the molecular mechanisms by which the *RS03155* gene affects apoptosis in the *in vivo* model, including its interactions with host cellular signaling pathways, have yet to be explored. Beyond initial assessments of infection and apoptosis, future research into the long-term effects of the *RS03155* gene on host immune responses and disease outcomes remains indispensable. Overall, in this study, the NbtS protein from *N. farcinica* activated the MAPK/NF-κB signaling pathway in microglial cells and induced apoptosis. These findings highlight the potential role of the NbtS protein in modulating immune responses and cell viability in the CNS, which could have implications for understanding and treating infections and inflammatory diseases in the brain.

### Conclusions

The present study showed that NbtS protein induces cell apoptosis in microglial cells. In addition, stimulation with NbtS protein also upregulates the MAPK/NF-κB p65 signaling pathway, leading to the expression of IL-1β and TNF-α. Our study underscores the critical role of the NbtS protein in the immune response and apoptosis triggered by *N. farcinica*. These findings suggest that targeting the NbtS protein could be a promising strategy for developing therapeutic interventions against *Nocardia* infections.

## MATERIALS AND METHODS

### Bacterial strains, cells, and mice

The standard *N. farcinica* strain IFM10152, sourced from the German Resource Centre for Biological Materials, was cultured in brain heart infusion (BHI) medium (Oxoid, China) under controlled conditions at 37℃. Our team constructed and maintained the *N. farcinica RS03155* mutant strain under the same conditions as the wild type. *Escherichia coli* strains DH5α and BL21(DE3) (Vazyme, China) were cultivated in Luria-Bertani (LB) broth (Oxoid) at a constant temperature of 37℃. All cell lines used in this study, including human microglial clone 3 (HMC-3) and mouse microglia cells (BV2), were purchased from the Cell Resource Center, Peking Union Medical College. HMC-3 and BV2 cells were cultured in Dulbecco’s modified Eagle medium (USA) supplemented with 10% fetal bovine serum (Gibco) at 37°C in a 5% CO_2_ humidified incubator. Balb/c mice were sourced from SPF Biotechnology, Beijing, China.

### Infection of mice with *N. farcinica* and *RS03155* mutant strain

Wild-type and mutant strains were cultured in BHI liquid medium until reaching logarithmic growth phase. The cultures were washed three times with phosphate-buffered saline (PBS) and adjusted to 1 × 10^8^ CFU/mL. Mice received intravenous injections of 5 × 10^6^ CFU of either parent or mutant bacterial strains. Mice’s body temperature and weight were continuously monitored for 7 dpi. At 1, 3, 7, and 14 dpi, 10 mice from each group were euthanized to collect and homogenize brain tissues. After serial dilutions, homogenates were plated on BHI agar for colony counting.

### Plasmids, antibodies, and reagents

The pET32a vector (in our laboratory) was utilized to express *N. farcinica* NbtS protein in *E. coli*. The following antibodies were purchased from Cell Signaling Technology (Danvers, USA): Phospho-MAPK Family Antibody Sampler Kit (9910), β-actin antibody (4967S), NF-κB p65 (D14E12) XP rabbit mAb (8242), caspase-9 (C9) mouse mAb (9508), Bax antibody (2772), Bcl-2 (D17C4) rabbit mAb (3498), cleaved caspase-3 (Asp175) antibody (9661), and IRAK4 antibody #4363. IRAK1 polyclonal antibody (10478-2-AP), MYD88 polyclonal antibody (23230-1-AP), and TLR4 monoclonal antibody (66350-1-Ig) were purchased from Proteintech Group, Inc. (Wuhan, China). Anti-pro-caspase-3 antibody (ab32499) was purchased from Abcam Inc. (Cambridge, UK). The endotoxin removal kit was obtained from GenScript (Nanjing, China). Human TNF-α, mouse TNF-α, and human IL-1β ELISA kits were used in this study (BD, USA), including mouse IL-1β from Thermo Fisher Scientific (Waltham, USA).

### Conservation analysis of the RS03155 gene

Conservation analysis of the RS03155 gene, encoding the NbtS protein, was performed. Initially, a BLAST analysis was conducted using the National Center for Biotechnology Information database (see Fig. S3 in the supplemental material). Subsequently, genomic DNA was extracted from randomly selected clinical strains of *N. farcinica* preserved in our laboratory to serve as PCR amplification templates. The RS03155 gene’s presence in each strain was verified via 1.5% agarose gel electrophoresis. Furthermore, antigenic epitopes of the RS03155 gene were predicted using the online website (http://tools.iedb.org/ellipro/).

### Cloning, expression, and purification of recombinant NbtS

*N. farcinica* NbtS was synthesized by Tsingke Biotech (Beijing, China), cloned into pET32a, and transformed into *E. coli* DH5α. Subsequently, *E. coli* DH5α was cultured on LB agar medium containing 100-µg/mL ampicillin (Solarbio, Beijing, China) to select positive clones. After plasmid sequencing, it was transferred to *E. coli* BL21 cells and cultured on LB plates with 100-µg/mL ampicillin to isolate single colonies. Colonies were cultured overnight at 37°C in LB liquid medium with ampicillin, followed by plasmid extraction and sequencing to identify recombinants. Recombinant *E. coli* BL21 cells were cultured at 37°C in LB medium containing 100-µg/mL ampicillin and induced with 0.2 mM IPTG (Solarbio) at 28°C overnight. Bacteria were ultrasonicated and then centrifuged at 12,000 revolutions per minute (rpm) for 20 min at 4°C. Pellet and supernatant were analyzed via SDS-PAGE. Recombinant proteins were then purified using the His·Bind purification kit (LABLEAD, China) according to the manufacturer’s instructions. Endotoxins were removed using the ToxinEraser Kit (GenScript) from purified recombinant NbtS preparations. Protein concentrations were measured using a bicinchoninic acid (BCA, Vazyme) assay and stored at −80°C until use.

### Preparation of *Nocardia* antiserum

*N. brasiliensis*, *N. farcinica*, *N. transvalensis*, *N. otitidiscaviaru*, *N. veterans*, *N. nova*, and *N. cyriacigeorgica* were cultivated in BHI liquid medium under conditions of 37°C and 180 rpm to the logarithmic growth phase. The bacterial culture was then centrifuged and rinsed with PBS three times, and the concentration of the bacterial suspension was adjusted to an optical density of 0.8. Mice were subcutaneously injected with 50 μL of the bacterial suspension, followed by two booster immunizations at 7-day intervals. A control group was administered the same volume of sterile PBS. On the seventh day after the third immunization, blood was gained from the eyeballs. After being allowed to stand at room temperature for 1 h, the blood was stored at 4°C for 5–6 h to facilitate clotting and retraction. Subsequently, the blood was centrifuged at 4,000 rpm for 10 min. The serum was then assayed for its antibody titer and stored at −80°C for subsequent use.

### Immunogenicity determination of the NbtS protein

The purified protein underwent SDS-PAGE and was transferred to a polyvinylidene fluoride (PVDF) membrane (Merck, Germany) at 15 V for 1 h using a semi-dry method. The membrane was incubated with 5% skim milk in Tris-buffered saline Tween(TBST) solution on a shaker at room temperature for 2 h, followed by three washes with TBST, each for 10 min. The membrane was then incubated overnight at 4°C with the following primary antisera: anti-*N*. *brasiliensis* whole-cell mouse serum (1:1,000), anti-*N*. *farcinica* whole-cell mouse serum (1:1,000), anti-*N*. *transvalensis* whole-cell mouse serum (1:1,000), anti-*N*. *cyriacigeorgica* whole-cell mouse serum (1:1,000), anti-*N*. *otitidiscaviarum* whole-cell mouse serum (1:1,000), anti-*N*. *veterans* whole-cell mouse serum (1:1,000), anti-*N*. *nova* whole-cell mouse serum (1:1,000), His-tag rabbit polyclonal antibody (1:4,000) (Biodragon, China), and control mouse serum (1:1,000). After three washes with TBST, the membrane was incubated for 1 h at room temperature with horseradish peroxidase-conjugated goat anti-mouse IgG secondary antibody (1:5,000) (Biodragon), followed by three washes with TBST prior to developing.

### CCK-8 experiment

Cell viability was assessed using a CCK-8 kit (Beyotime Biotechnology, China) as per the manufacturer’s instructions. BV2 and HMC-3 cells were seeded in 96-well plates at 10,000 cells/well. Once grown to 90% confluence, cells were washed twice with PBS and exposed to varying concentrations of NbtS protein for 24 h. Ten-microliter CCK-8 solution was added to each well, and the absorbance at 450 nm was measured using a Multiskan GO full-wavelength microplate reader (Thermo Fisher Scientific). Cell viability was calculated by the protocol calculation formula.

### Cytotoxicity assay

BV2 and HMC-3 cells were seeded at a density of 10,000 cells/well in a 96-well plate and cultured for 16–18 h. Cells were then infected with *N. farcinica* wild-type and RS03155 deletion strains at multiplicities of infection of 5 and 10. Cell cytotoxicity was assessed using an LDH cytotoxicity assay kit (Beyotime Biotechnology), following the manufacturer’s instructions.

### Histopathology

Brain tissues from wild-type *N. farcinica*, RS03155 mutant deleted strain, and PBS control infection mice were fixed in 4% paraformaldehyde and embedded in paraffin. Samples were cut into 5-mm sections, stained with hematoxylin and eosin, and then imaged using a biomicroscope (Eclipse Ci-L; Nikon, Japan) according to the manufacturer’s instructions. Pathological analysis was conducted on the images.

### Cytokine ELISAs

BV2 and HMC-3 cells were seeded at a density of 2 × 10^5^ cells/well in 24-well plates and stimulated with NbtS or LPS (Sigma-Aldrich, USA) for 18 h. To ensure residual LPS in the NbtS preparation did not induce immune cell stimulation, NbtS was pre-incubated with Polymyxin B (Sigma-Aldrich), a specific inhibitor of LPS. Subsequently, cell culture supernatants were collected and TNF-α and IL-1β levels were measured using ELISA. Brain tissues from mouse infection experiments were also collected, centrifuged at 12,000 rpm for 10 min, and analyzed by ELISA.

### TUNEL assay

Sterile cell coverslips were placed in 24-well plates, and BV2 cells were seeded at a density of 2 × 10^5^ cells/well and then incubated at 37°C for 16–18 h. BV2 cells were then treated with wild-type *N. farcinica*, RS03155 mutant strain, NbtS, and LPS for 18 h, fixed in 4% paraformaldehyde for 30 min, and permeabilized with 0.3% Triton X-100 in PBS for 5 min at room temperature. Cell apoptosis was detected using a one-step TUNEL apoptosis assay kit (green fluorescence) (Beyotime Biotechnology) according to the manufacturer’s instructions. Cell coverslips were then mounted on slides and sealed with anti-fade mounting medium (Solarbio) for observation under a fluorescence microscope. Brain tissue from mice infected with the wild-type strain of *N. farcincia*, the RS03155 knockout strain, and the control group was similarly evaluated for cellular apoptosis using TUNEL staining (ServiceBio, Hubei, China).

### Quantitative real-time PCR analysis

BV2 and HMC-3 cells were seeded at a density of 8 × 10^5^ cells/well in six-well plates overnight. To inhibit MAPK and NF-κB signaling, cells were pre-treated with p38 (10 µM), ERK (20 µM), JNK (20 µM), and NF-κB (5 µM) inhibitors at 37°C for 1 h prior to NbtS treatment. Total RNA was extracted from cells using the TRIzol and chloroform method. Genomic DNA contamination was eliminated and cDNA was synthesized using the FastKing One-Step gDNA Removal and First Strand cDNA Synthesis SuperMix Kit (TIANGEN, China). Quantitative real-time PCR was performed with the SuperReal PreMix Plus (SYBR Green) reagent (TIANGEN) as per the manufacturer’s protocol. RNA levels of the analyzed genes were normalized to β-actin levels in each sample. Primers for TNF-α, IL-β, and β-actin (see Table S1 in the supplemental material) were synthesized by Tsingke Biotech. Experiments were conducted in triplicate.

### Western blot analysis

HMC-3 and BV2 cells were seeded at a density of 8 × 10^5^ cells/well into six-well plates and stimulated with NbtS for varying durations. Cells were lysed with RIPA Lysis Buffer (Strong) (CWBIO, China) supplemented with phosphatase and protease inhibitors (CWBIO) on ice for 20 min. After centrifugation at 14,000 × *g* for 10 min, the supernatant was collected for protein concentration determination using the BCA Protein Assay Kit (Vazyme). Equal amounts of protein were separated by SDS-PAGE and then transferred onto PVDF membranes (Millipore). After blocking with QuickBlock Blocking Buffer (Beyotime Biotechnology) for 15 min, PVDF membranes were incubated overnight at 4°C with primary antibodies (p-ERK1/2, p-p38, p-JNK, p-p65, Bcl2, Bax, pro-caspase-3, caspase-9, cleaved caspase-3, and β-actin). The membranes were then incubated with horseradish peroxidase-conjugated anti-rabbit IgG (CST, USA) or anti-mouse IgG (Biodragon) for 1 h and visualized using Clarity Max Western ECL Substrate (Bio-Rad, USA).

### Statistical analysis

Data analysis and visualization were performed using MATLAB software (MathWorks, Natick, MA, USA). Statistical outcomes were presented as mean ± standard deviation. Student’s *t*-test was applied to compare each experimental group with the control group, assuming homogeneity of variance and normal distribution. Significant differences between the control group and experimental groups were denoted by “*” (*P* < 0.05) and “**” (*P* < 0.01).

## SUPPLEMENTAL MATERIAL

Supplemental material msystems.00893-24-s0001.docx Supplemental figures and table.

ASM does not own the copyrights to Supplemental Material that may be linked to, or accessed through, an article. The authors have granted ASM a non-exclusive, world-wide license to publish the Supplemental Material files. Please contact the corresponding author directly for reuse.
