# Supplementary material for: Activation of NF-κB/MAPK signaling and induction of apoptosis by salicylate synthase NbtS in Nocardia farcinica promotes neuroinflammation development
Source: mSystems. 2024 Sep 6;9(10):e00893-24. doi: 10.1128/msystems.00893-24 (PMC11494946; doi:10.1128/msystems.00893-24)
Supplement: Supplemental material — Supplemental figures and table. [file msystems.00893-24-s0001.docx]

Supplemental table1 qPCR primer sequence

| Primers | sequence（5’-3’） |
| --- | --- |
| β-actin F  β-actin R | TATAAAACCCGGCGGCGCA  GTCATCCATGGCGAACTGGTG |
| TNF-α F  TNF-α R  IL-1β F  IL-1β R | CGCTCTTCTGTCTACTGAACTTCGG  GTGGTTTGTGAGTGTGAGGGTCTG  TGCCACCTTTTGACAGTGATG  ATGTGCTGCTGCGAGATTTG |


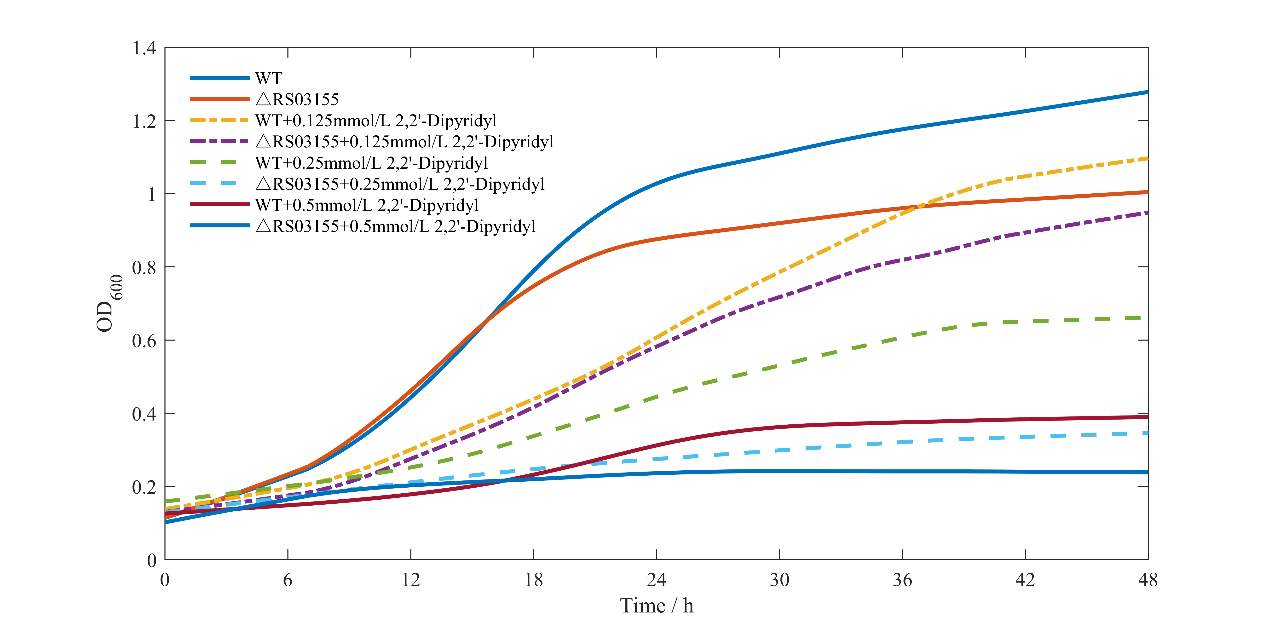


Fig. S1 Using 2,2'-bipyridyl as an iron chelator, we investigated the growth of wild-type and RS03155 knockout strains of *Nocardia* *farcinica* under varying concentrations of 2,2'-Bipyridyl in Brain Heart Infusion (BHI) medium.


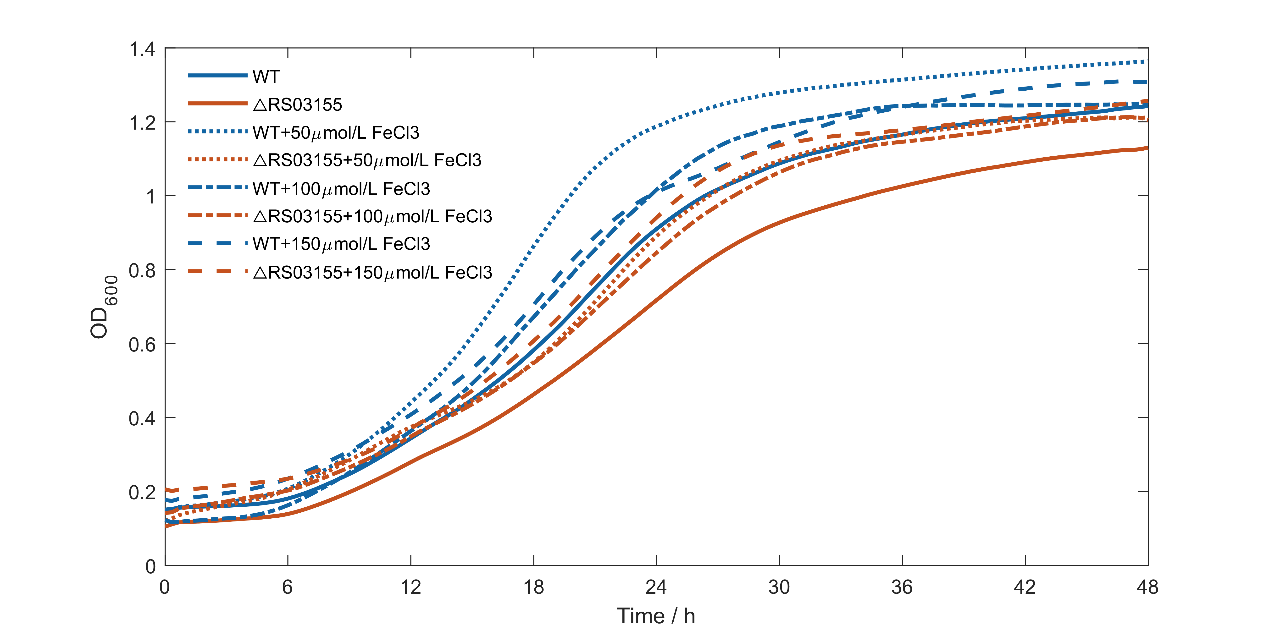


Fig. S2 Additional supplementation of iron at different concentrations in BHI medium to study the growth of wild-type and RS03155 knockout strains of *Nocardia farcinica*.


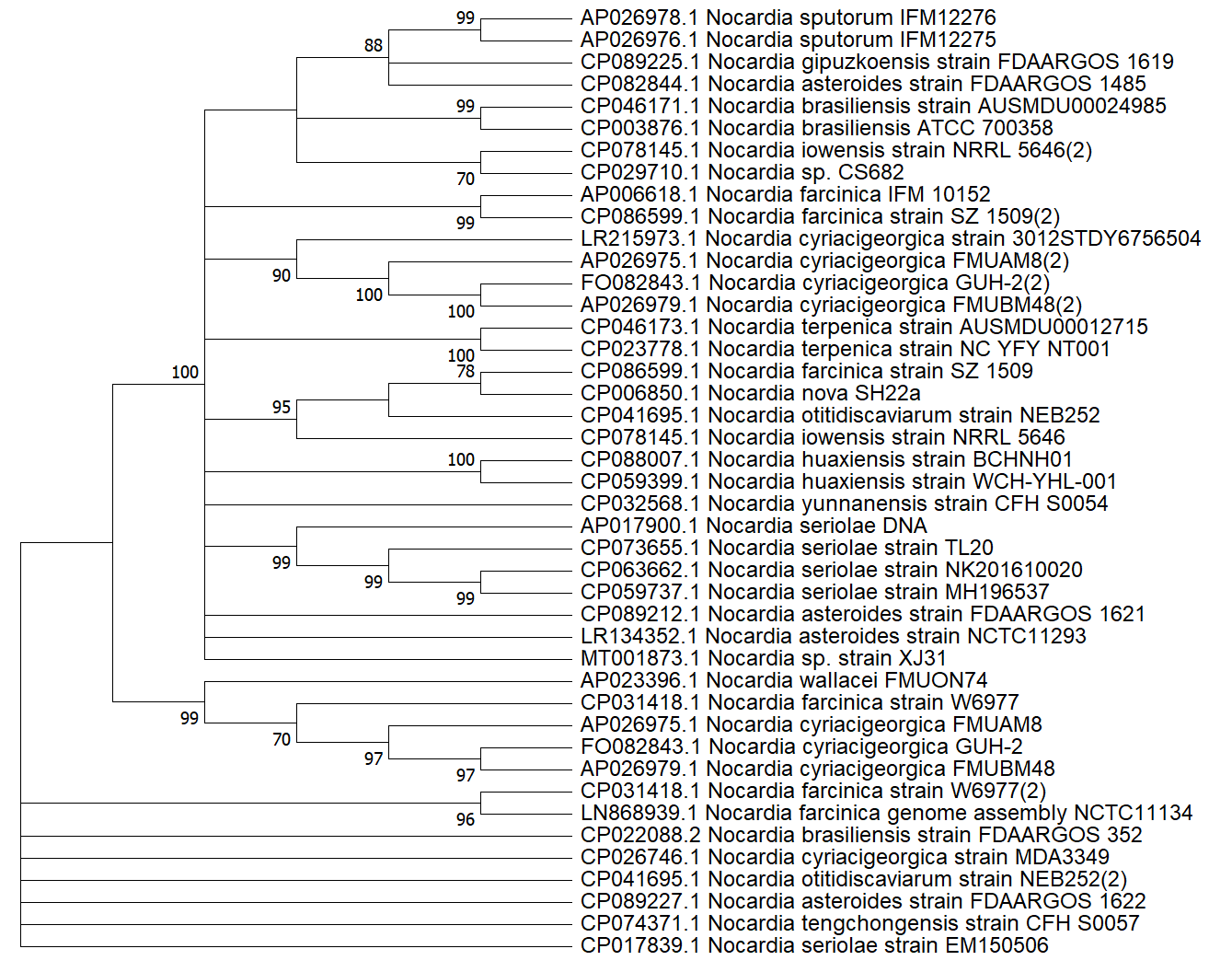


Fig. S3 The RS03155 gene sequence was compared by BLAST to construct a phylogenetic tree
